# Ipsilesional arm training in severe stroke to improve functional independence (IPSI): phase II protocol

**DOI:** 10.1186/s12883-022-02643-z

**Published:** 2022-04-12

**Authors:** Candice Maenza, Robert L. Sainburg, Rini Varghese, Brooke Dexheimer, Marika Demers, Lauri Bishop, Shanie A. L. Jayasinghe, David A. Wagstaff, Carolee Winstein, For the IPSI Investigative Team

**Affiliations:** 1 Department of Neurology, Pennsylvania State University College of Medicine, 500 University Drive, Hershey, PA 17033 USA; 2 Department of Kinesiology, Pennsylvania State University, 27 Rec Hall, University Park, PA 16802 USA; 3 Division of Biokinesiology and Physical Therapy, Herman Ostrow School of Dentistry, University of Southern California, Los Angeles, CA USA; 4 Department of Human Development and Family Studies, Pennsylvania State University, 102 HHD Building, University Park, PA 16802 USA; 5 Department of Neurology, Keck School of Medicine, University of Southern California, Los Angeles, CA USA

**Keywords:** Ipsilesional deficits, Ipsilateral deficits, Stroke remediation, Stroke rehabilitation, Arm training, Virtual reality training, Less-impaired arm, Stroke motor deficits

## Abstract

**Background:**

We previously characterized hemisphere-specific motor control deficits in the ipsilesional, less-impaired arm of unilaterally lesioned stroke survivors. Our preliminary data indicate these deficits are substantial and functionally limiting in patients with severe paresis.

**Methods:**

We have designed an intervention (“IPSI”) to remediate the hemisphere-specific deficits in the ipsilesional arm, using a virtual-reality platform, followed by manipulation training with a variety of real objects, designed to facilitate generalization and transfer to functional behaviors encountered in the natural environment. This is a 2-site (primary site – Penn State College of Medicine, secondary site – University of Southern California), two-group randomized intervention with an experimental group, which receives unilateral training of the ipsilesional arm throughout 3 one-hour sessions per week for 5 weeks, through our Virtual Reality and Manipulation Training (VRMT) protocol. Our control group receives a conventional intervention on the contralesional arm, 3 one-hour sessions per week for 5 weeks, guided by recently released practice guidelines for upper limb rehabilitation in adult stroke. The study aims to include a total of 120 stroke survivors (60 per group) whose stroke was in the territory of the middle cerebral artery (MCA) resulting in severe upper-extremity motor impairments. Outcome measures (Primary: Jebsen-Taylor Hand Function Test, Fugl-Meyer Assessment, Abilhand, Barthel Index) are assessed at five evaluation points: Baseline 1, Baseline 2, immediate post-intervention (primary endpoint), and 3-weeks (short-term retention) and 6-months post-intervention (long-term retention). We hypothesize that both groups will improve performance of the targeted arm, but that the ipsilesional arm remediation group will show greater improvements in functional independence.

**Discussion:**

The results of this study are expected to inform upper limb evaluation and treatment to consider ipsilesional arm function, as part of a comprehensive physical rehabilitation strategy that includes evaluation and remediation of both arms.

**Trial Registration:**

This study is registered with ClinicalTrials.gov (Registration ID: NCT03634397; date of registration: 08/16/2018).

## Background

It is well understood that damage to one side of the brain, due to middle cerebral artery ischemic stroke, can lead to paresis, including movement limiting deficits in muscle tone and abnormal movement synergies in the arm and leg on the opposite side of the body to the brain lesion [1–4]. These impairments in the paretic contralesional arm and hand are targeted by most current clinical approaches to remediate upper limb function following stroke. It has been known for some time that sensorimotor deficits are also evident in the ipsilesional arm, though they are more subtle in expression than in the contralesional arm. Previous research has shown that such ipsilesional motor deficits can substantially limit functional independence [5–13]. Although these deficits appear mild, when compared to contralesional arm paresis, they can produce functional loss of dexterity for performance of activities of daily living [5, 6, 14–17], and are associated with deficits in movement coordination and accuracy, as measured by motion tracking and kinematic analyses [9, 14, 18]. Such ipsilesional arm motor deficits have recently been shown to vary with the severity of impairment of the contralesional paretic arm in chronic stroke survivors [5]. This means that stroke survivors with severe contralesional arm impairments will likely have more substantial motor deficits in the less-affected, ipsilesional arm. The severely paretic upper limb is essentially non-functional for most manipulation tasks, which leaves these stroke survivors dependent on the less-affected arm as the primary or sole manipulator for most activities of daily living. However, ipsilesional motor deficits can make even simple tasks laborious, time-consuming, and in some cases impossible to perform. Thus, in stroke survivors with severe contralesional arm paresis, intervention focused on remediating deficits in the ipsilesional arm is likely to improve functional independence, a hypothesis supported by recent pilot study findings from our laboratory [13].

Over two decades of research from our laboratories [3, 9, 19] and from other laboratories [16, 20] have established that arm movements recruit both hemispheres for motor control and coordination. This evidence has led to the development of a bihemispheric model of motor control, in which each hemisphere contributes different aspects of control to each arm. Most of these studies have enrolled right handers, and have shown that the left hemisphere mediates movement features that are measured early in movement and may reflect predictive aspects of control, such as time to peak velocity and speed, [9, 14, 20] initial accuracy of movement direction, as well as trajectory quality [21]. In contrast, the right hemisphere (in right handers) appears to mediate features of movement measured later in the course of the trajectory, including duration of the deceleration phase [14], final position stability and accuracy, as well as trajectory stability during the course of movement [22–26]. Simulation studies have demonstrated that these apparent lateralized movement features can be attributed to hybrid control, in which optimal trajectory planning and positional impedance control are mediated by different hemispheres, combined for the control of each arm [24, 27]. Behavioral asymmetries appear to reflect the specializations of the hemisphere contralesional to the arm in question. Some evidence suggests that the mirror-imaged specializations are expressed in left-handers [28]. This model has explained hemisphere-specific deficits that occur following right or left hemisphere damage, following stroke and importantly that differ, depending on the side of brain damage [3, 5, 29, 30].

Despite this body of research, remedial physical rehabilitation following stroke continues to focus primarily on the more obvious contralesional extremity deficits and tends to neglect the presence of ipsilesional arm motor deficits [31]. Therefore, when the less-affected ipsilesional arm is included in rehabilitation protocols, it is most often used for compensatory purposes or to assist the contralesional arm during bilateral tasks. In either case, potential motor deficits in the ipsilesional arm are not assessed, nor addressed as a remedial target for physical rehabilitation. We now seek to investigate the effects of remedial training of the ipsilesional limb on arm function and on functional independence following unilateral stroke. On the basis of evidence established in previous mechanistic studies, we designed movement-based training programs for the ipsilesional arm that differentiate right and left hemisphere-mediated features of control so as to specifically target hemisphere-specific deficits in individuals with left and right hemisphere damage. We recently published results of a pilot study that provided support for hemisphere-specific training of the ipsilesional limb to improve ipsilesional arm function and functional independence [13]. This intervention study included 13 participants with chronic stroke that had both functionally limiting ipsilesional upper-extremity impairments and moderate to severe contralesional upper-extremity impairments. Following 3 weeks of training, 3 times per week, for 1.5 h per session, participants showed improvements in ipsilesional arm dexterity that generalized to substantially improve functional independence, as measured by the motor components of the Functional Independence Measure (FIM) [32]. We now modify and extend the pilot study protocol to a sufficiently powered, two-site randomized control phase II clinical trial designed to target hemisphere specific deficits of the ipsilesional arm. Two groups of stroke survivors whose stroke was in the territory of the middle cerebral artery (MCA) and with severe contralesional paresis receive either hemisphere-specific remedial training of the ipsilesional arm or conventional motor remediation of the contralesional arm. We hypothesize that both groups will improve performance of the targeted arm, but that the ipsilesional arm remediation group will show greater improvements in functional independence. The results of this study are expected to inform upper limb evaluation and treatment to consider ipsilesional arm function, as part of a comprehensive physical rehabilitation strategy that includes evaluation and remediation of both arms.

## Methods/design

One hundred twenty chronic stroke survivors with severe motor impairments are expected to be recruited into a two-group parallel, randomized control trial involving two dose-matched 5-week interventions. The experimental intervention focuses on the ipsilesional limb using Virtual Reality and Manipulation Training (VRMT), which includes tasks that target hemisphere-specific deficits that have been shown to result from left or right hemisphere damage. The control intervention targets the contralesional (paretic) limb and involves conventional therapies. Primary outcome measures are intended to assess sensorimotor impairments and activity limitations over short and long retention intervals following completion of the intervention. The overarching goal of this phase II clinical intervention trial is to provide evidence that can be used to optimize rehabilitation therapies for chronic stroke survivors with severe upper limb paresis and maximize the recovery of functional performance and activities in these individuals. Written informed consent is obtained from all participants at their first study visit. All documentation, procedures, and modifications for the trial are approved by Institutional Review Boards of the 2 participating sites: Penn State College of Medicine (STUDY00008385) and the University of Southern California (HS-18–00,802), with the proviso that for this protocol, the USC site is ceded to the IRB of Record at the primary site, Penn State College of Medicine.

### Type of design

**Fig. 1:**
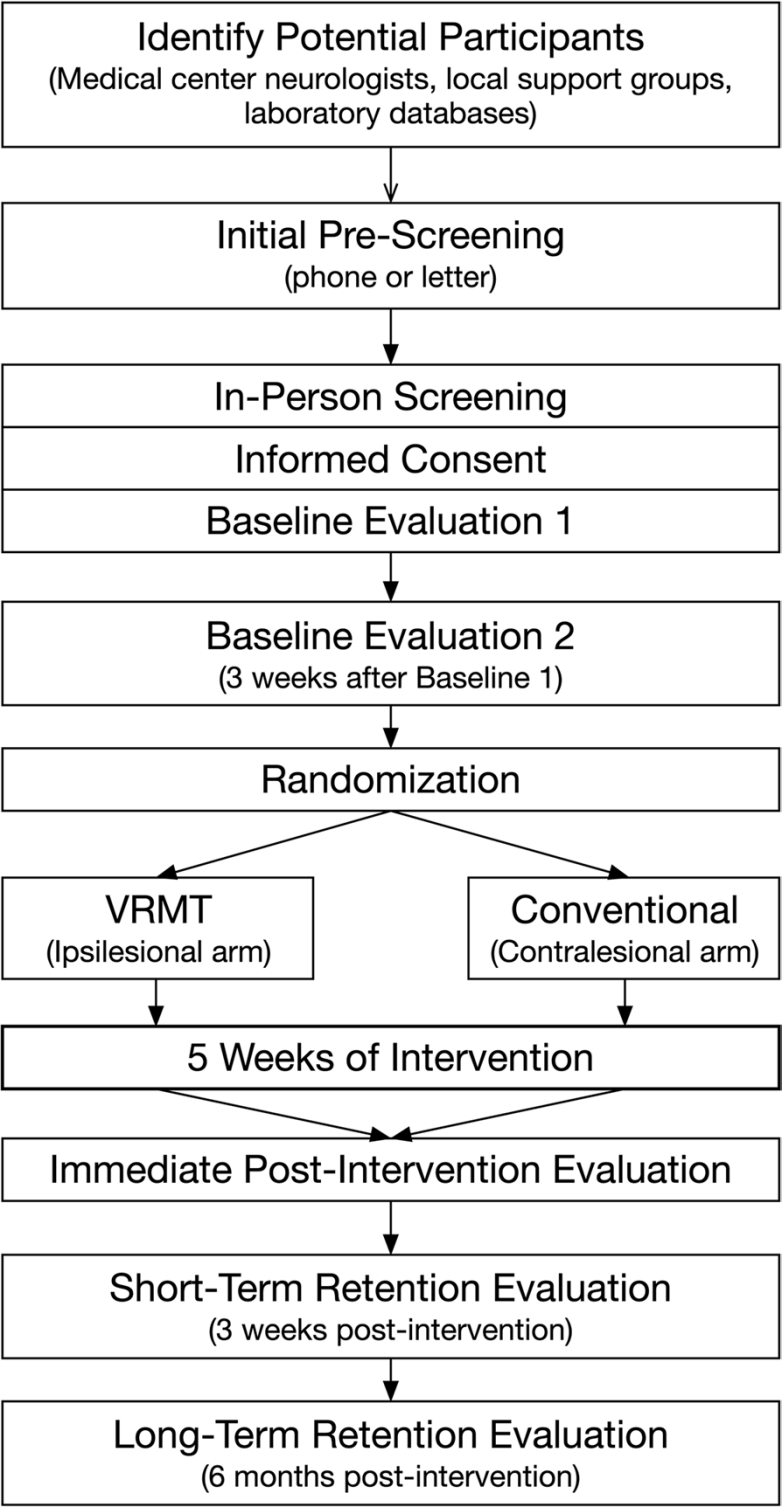
Study flow diagram from first referral to final follow-up evaluation

The IPSI trial is a two-arm, parallel-group, randomized, single-blind intervention study of an upper-extremity rehabilitation intervention tailored to address hemisphere-specific deficits of the ipsilesional arm. Chronic stroke survivors are recruited through a combination of existing patient databases, local support groups, and medical center neurologists across the two sites (primary site – Penn State College of Medicine, secondary site – University of Southern California). Primary and secondary outcome measures are recorded initially at baseline, immediately post-intervention, 3-weeks (short-term retention) and 6-months (long-term retention) post-intervention. Trained and standardized assessors are blinded to group assignment. If an assessor becomes blinded at any time, an alternate assessor will score the evaluation session outcome measures. A complete diagram of the study flow is shown in Fig. 1. See sections below for details about the end points and outcome measures.

### Study enrollment

Both sites are expected to randomize 60 individuals over the duration of the study, for a total of 120 participants over a four-year recruitment period. Table 1 outlines the screening process we are using to determine eligibility, including a phone pre-screen and two in-person visits separated by 3 weeks. Participants who complete the screening process and meet eligibility requirements are randomized to one of the two intervention arms. This study is currently enrolling participants and is expected to continue until 2024.

**Table 1:** Study phase flow up to point of randomization

Phase	Event	Purpose	Time Interval
Pre-Screening	Chart Review	A search of local stroke databases at each site to determine potentially eligible participants	
Screening	Participant Eligibility Criteria Checklist	An initial brief questionnaire delivered over the phone	
Study Informed Consent	Consent for Research Document	Explanation of the study in greater detail and signature by the participant expressing intent to proceed with the study	In-person visit #1
Baseline 1 Screening	Clinical Evaluation	Evaluations that do not contribute to eligibility and are only assessed at Baseline 1 screening include: handedness (Edinburgh Handedness Inventory), visual neglect (line bisection test), apraxia Clinical and kinematic outcome measures are assessed	In-person visit #1
Research MRI	MRI	MRI^a^	Any time prior to starting intervention
Randomization	Group Assignment	Randomization of eligible participant to one of the two intervention arms	Immediately following in-person visit #1
Baseline 2 Screening	Clinical Evaluation	Clinical and kinematic outcome measures are assessed to establish consistency with baseline 1 screening	In-person visit #2

^a^If participant cannot receive an MRI for any reason, medical records will be used to confirm participants have a unilateral MCA brain lesion

### Accessing and collecting personal health information

Prior to recruitment, members of the study team review potential participants’ medical records (when available) to assess whether potential participants meet eligibility criteria related to medical history (see Table 2: Eligibility criteria).

**Table 2:** Eligibility criteria

**Inclusion**
1. Neuroradiological confirmation of unilateral stroke confirmed by a review of their medical record and/or a research grade MRI scan
2. Fugl-Meyer Upper Extremity Assessment (FMA) score 0–28 and 0 on mass extension and prehension
3. Ipsilesional arm performance: JTHFT total score > 70 s OR score of > 45 s (LHD) OR > 40 s (RHD) excluding the writing task^a^
4. > 6 months post stroke
5. Demonstrates cognitive abilities
6 Either R- or L-handed^b^
**Exclusion**
1. Neurological confirmation of concomitant damage to cerebellum, brain stem or significant white matter changes
2. Bilateral stroke
3. History of neurological disease other than stroke (e.g., Parkinson’s Disease, head trauma)
4. History of a major psychiatric diagnosis (e.g., schizophrenia, major affective disorder)
5. Prior hospitalization for substance abuse
6. Peripheral disorders significantly affecting sensation or movement of the arms (e.g., pain, arthritis)
7. Currently taking prescription medications with known sedative properties that could interfere with sensorimotor function
8. Activity limiting joint pain

^a^ Updated in February 2019 to differentiate LHD/RHD requirements

^b^ Updated in July 2021 to include left handed participants

### Screening process

The screening process has two phases: 1) Pre-screen using IRB approved participant databases and a self-report health-screening phone questionnaire, and 2) In-person screen of individuals who pass the pre-screen. Both sites use advertisements and local stroke support group networks to recruit patients. At USC, recruitment primarily occurs by contacting individuals previously enrolled in an IRB approved Registry for Aging and Rehabilitation Evaluation (RARE) database. At PSU, a database of previously admitted stroke patients at Penn State Hershey Medical Center (PSHMC) is provided to the team on a quarterly basis. The pre-screen includes review of patient medical records and/or a phone screen questionnaire. If the potential participant passes pre-screening, s/he is scheduled for an in-person screening session at either USC or PSU (Baseline 1) where written informed consent is obtained, the eligibility checklist is reviewed, and all clinical and kinematic outcome measures are assessed. We also determine the level of apraxia using a standardized test for ideomotor limb apraxia [33] and hemispatial neglect using the line bisection test [34]. Neither of these conditions are exclusionary, but will be used in pre-planned secondary analyses to determine whether either of these conditions moderates the intervention’s impact on performance. Individuals return 3 weeks after Baseline 1 for a second baseline (Baseline 2) in which the primary and secondary outcome measures are repeated to establish consistency and stability with Baseline 1, which will be assessed in post-hoc analysis.

### Inclusion and exclusion criteria

Participants include adults with a radiologically confirmed diagnosis of unilateral ischemic or hemorrhagic stroke in the distribution of the middle cerebral artery, severe contralesional arm paresis, and a threshold measure of ipsilesional arm impairment. A comprehensive list of inclusion and exclusion criteria are outlined in Table 2. Any concomitant care is documented, but is not exclusionary.

### Modifications to the inclusion criteria

Since February 2019 when we enrolled our first participant, we saw a need to modify the original inclusion criteria for threshold impairment in the ipsilesional arm, as measured by the Jebsen-Taylor Hand Function Test (JTHFT). Originally, we had specified an overall score of 70 s on the JTHFT, as the threshold for inclusion, regardless of hemisphere of damage and arm. However, the dominant and non-dominant arms perform differently on the JTHFT in age matched controls, on both non-writing tasks and writing tasks, and thus we expanded our threshold for inclusion, as follows: After removing the writing task, a score > 45 s for left hemisphere damage (LHD) or > 40 s for right hemisphere damage (RHD) on the JTHFT indicates substantial ipsilesional deficits, and thus meets the ipsilesional hand eligibility for study participation. These values were derived from previous data from our laboratory in older participants without neurological deficits. In addition, in July 2021 after post-COVID restart, we again modified our inclusion criteria to include left handers (previously excluded) in order to expand our recruitment pool. In the case of left-handers, the criteria for inclusion based on JTHFT is mirror imaged, such that a score > 45 s for RHD or > 40 s for LHD on the JTHFT indicates substantial ipsilesional arm deficits.

### Endpoints and outcome measures

**Fig. 2:**
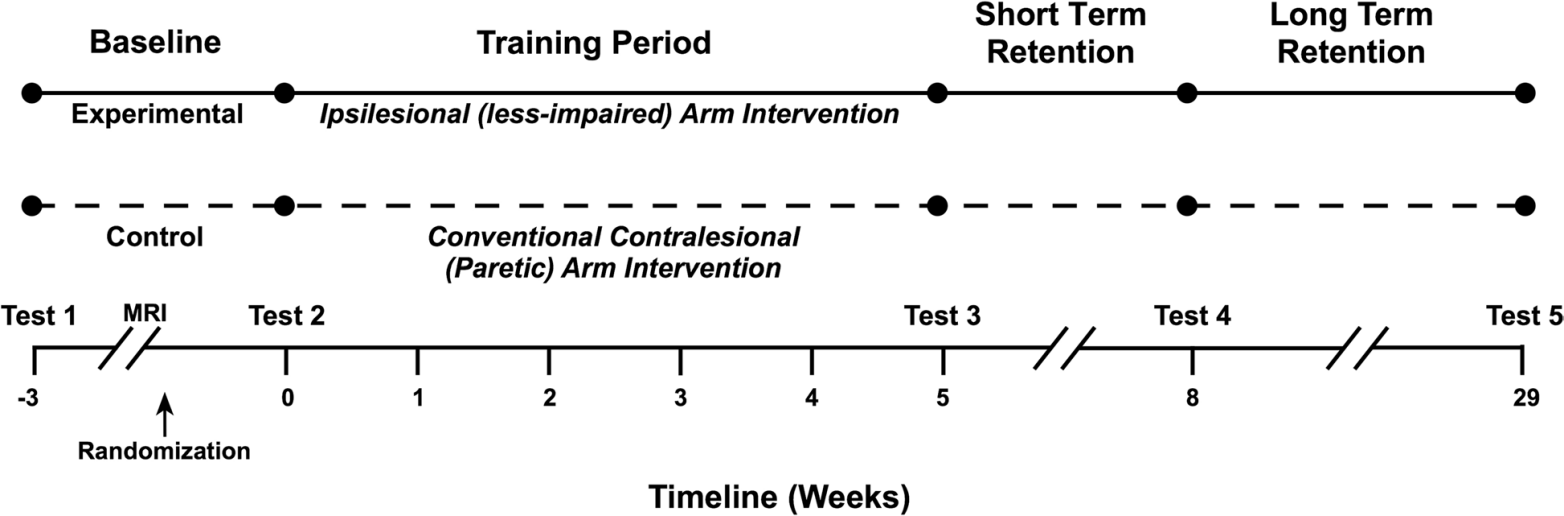
Timeline of study events for each participant

There are five evaluation points throughout the study: Baseline 1, Baseline 2, immediate post-intervention, and 3-weeks (short-term retention) and 6-months post-intervention (long-term retention) (Fig. 2). The immediate post-intervention evaluation is the primary endpoint that will be used to test the primary and secondary hypotheses (see Table 3 for time points at which each assessment is performed).

**Table 3:** Assessments at baseline and follow-up evaluation sessions

Assessment	Baseline 1	Baseline 2	Post-Intervention^c^	3 weeks Post-Intervention^d^	6-months Post-Intervention^d^	Contralateral Arm	Ipsilesional Arm
JTHFT^a^	X	X	X	X	X		X
FMA^a^	X	X	X	X	X	X	
Barthel Index^a^	X	X	X	X	X		
ABILHAND^a^	X	X	X	X	X		
Position Variability^b^	X	X	X	X	X		X
Work Space Area^b^	X	X	X	X	X	X	
FIM^b^	X	X	X	X	X		
Apraxia Battery	X						X
Line Bisection Test	X						X
Edinburgh Handedness Inventory	X						

^a^ indicates primary outcome measure; ^b^ indicates secondary outcome measure; ^c^ indicates primary endpoint; ^d^ indicates secondary endpoint

Licensed occupational/physical therapists or research staff, who have been trained and standardized for evaluations, are blinded to group assignments and standardized for administration perform all evaluations. The FMA and apraxia battery are filmed and scored offline using a digital video camera and then uploaded to a HIPAA-compliant shared server. All evaluations are scored by the evaluator and are then entered onto Penn State University’s Research Electronic Data Capture (REDCap) within one week of acquisition. Each entry is then double-checked for accuracy each month by an additional lab member.

### Primary outcome measures

#### Jebsen-Taylor Hand Function Test (JTHFT)

The JTHFT is a valid and reliable measure of manual dexterity [35, 36]. The assessment includes seven subtests: writing, flipping cards, picking up small objects, simulating feeding, stacking checkers, picking up light objects, and picking up heavy objects. Each participant is instructed to perform each subtest as quickly as they are able; completion time for each subtest is recorded. The psychometric properties of the JTHFT have been established in the adult and older adult populations [36].

#### Fugl-Meyer Upper Extremity Assessment (FMA)

The FMA is a widely used, valid and reliable measure of sensorimotor impairment of the upper limbs and functional mobility in stroke survivors [37–39]. The upper extremity motor function domain evaluates the ability to make upper limb movements in and out of synergy patterns. It consists of 33 items that evaluate finger, hand, and arm movements, grasp, reflex action, and coordination graded on a 3-point ordinal scale (from 0 = cannot perform, 1 = performs partially, 2 = performs fully). The total score for the upper extremity motor function domain is 66, with lower scores indicating greater motor impairment. The minimal clinically important difference (MCID) for the FMA is considered to be approximately 10% of the maximum score or 6.6 points [40].

### Barthel index

The Barthel Index is a valid self-report measure of functional independence in the domains of personal care and mobility in individuals with chronic, disabling conditions [41, 42]. It consists of 10 items, with higher weight placed on some items over others. Bathing and grooming are given a score of either 0 (unable to perform) or 5 (fully independent). Feeding, dressing, bowel control, bladder control, toilet use, and stair climbing are given a 0 (unable), 5 (needs assistance), or 10 (independent). Chair transfer and ambulation are scored with either a 0 (unable), 5 (major help needed, including wheelchair), 10 (minor help needed), or 15 (independent). The index also indicates the need for assistance in care. The total possible score is 100, with higher scores indicating greater degrees of functional independence.

### ABILHAND questionnaire

The ABILHAND is a reliable interview-based questionnaire of bimanual ability for adults with upper limb impairments [43]. It has been validated in chronic stroke, along with other chronic motor impairment conditions [44, 45]. It includes 23 items ordered from most difficult to least with each item scored on a three-level response scale (impossible: 0, difficult: 1, or easy: 2).

### Secondary outcome measures

#### Functional Independence Measure (FIM)—Motor

The FIM is a reliable evaluation of a person’s burden of care based on the level of assistance they require for a range of motor-based activities of daily living [46]. The FIM consists of 18 items including both motor and cognitive components. For the purposes of this study, only the 6 motor self-care tasks (motor subscale) are evaluated. Each of these items is scored on a Likert scale of one (maximal assistance) to seven (complete independence) for a total of 42 points.

### Kinematic evaluation

#### Equipment/apparatus

**Fig. 3:**
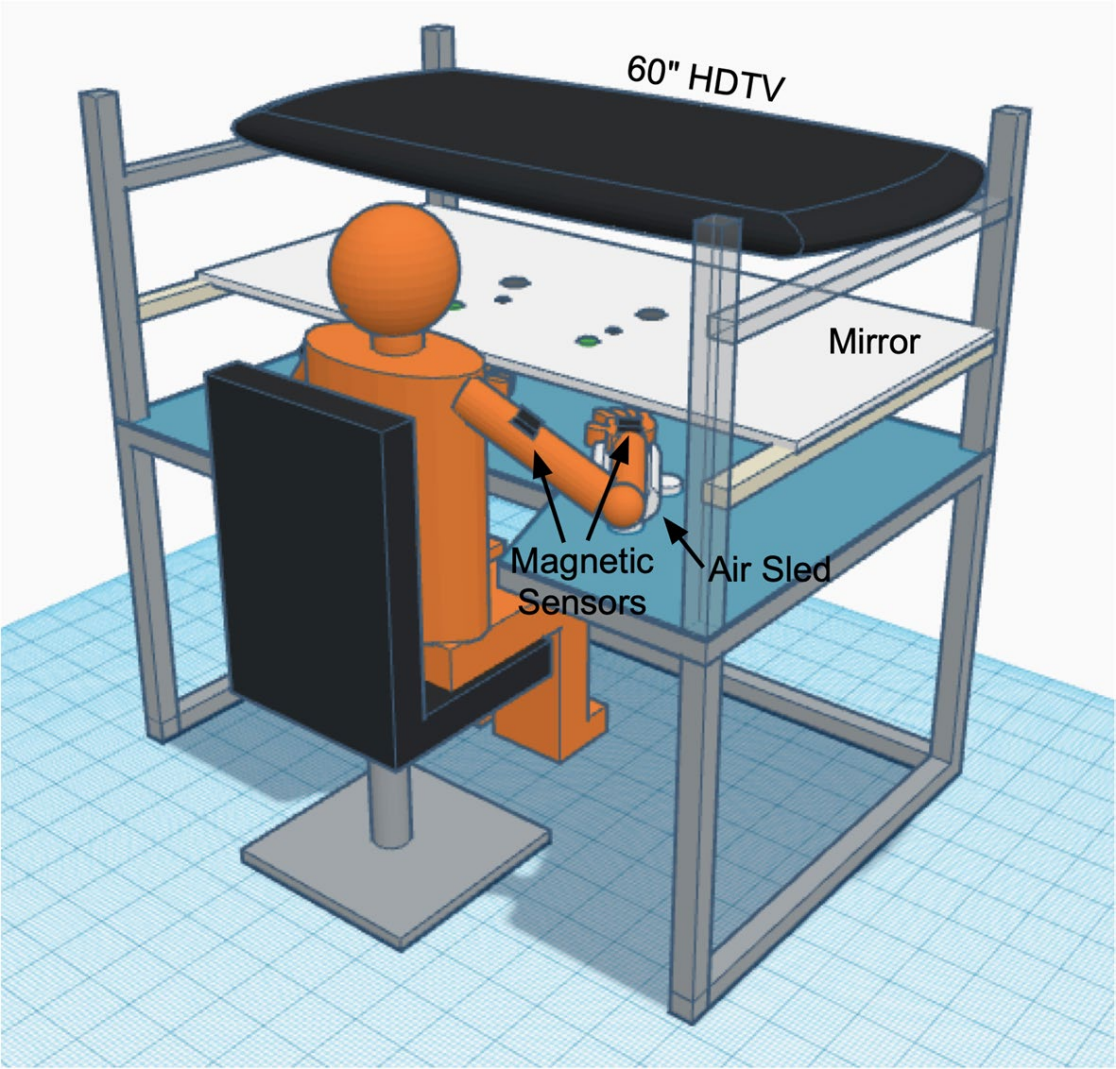
Kinematic-virtual reality set-up

Our kinematic-Virtual Reality set-up, depicted in Fig. 3, shows an individual seated in front of a table. This custom system (Kinereach®: designed and programmed by Sainburg) is used for kinematic testing as well as for Virtual Reality training of specific movement components. Each site’s laboratory has an identical system installed. For arm-supported tasks (2D), the forearms rest on air-cushion sleds that support the arms against gravity and nearly eliminate friction. For 3D tasks, the arm is held above the table-top, and a cursor, representing hand position, can only be seen when the arm is maintained off the tabletop. Task and movement feedback is displayed on a horizontal mirror positioned 35 cm above the table surface. This mirror reflects the stimuli presented on a horizontal, inverted, 60″ HDTV display. The first proximal interphalangeal joint of the hand reflects the position of the cursor. Six degrees of freedom Trackstar® magnetic sensors are attached to the limbs, while positions of bony landmarks are used to digitize the hand, forearm, and upper arm segments, allowing calculation of 10 degrees of freedom per arm, recorded at 116 Hz. Data are low-pass filtered using a 12 Hz zero lag Butterworth filter, prior to differentiating to yield velocity and acceleration profiles. Testing of the less-impaired arm (i.e. ipsilesional) kinematics is done during arm-supported (2D) tasks in order to be consistent with our previous studies of the less-impaired arm motor control deficits, while functional training of the less-impaired arm is done in 3D.

### Kinematic outcome measures

The kinematic evaluation is performed with the Kinereach system and includes two outcomes: the work space area and position variability. The work space area is a kinematic measure of the active range of motion of the paretic arm. It is computed as the total area circumscribed by the contralesional (paretic) hand on a horizontal surface. The position variability is a measure of kinematic variability in reaching movements, early and late in the movement. Specifically, the variance of the hand position is computed at peak velocity (early variance) and at the end of a targeted reaching movement (late variance).

### Outcome measures used to test study hypotheses

The change in JTHFT, FMA, ABILHAND and Barthel Index scores from baseline scores at 3 time points: immediately following training (primary endpoint), short-term, and long-term retention (secondary endpoints) are the primary outcome measures for determining efficacy of the intervention. The Functional Independence Measure (FIM) motor scale and kinematic measures of work space area and position variability constitute secondary outcome measures.

### Medication information

We collect data on each participant’s medication prescriptions and usage during pre-screening, during the screening process, prior to the first baseline evaluation, and at each follow-up visit after the intervention. A detailed list of medication type and dosage is recorded at Baseline 1. This list is queried and modified at each of the intervention sessions if necessary. Discontinued medications are also noted, as well as the addition of new medications. The medication data are intended to provide additional information about the medical status of participants.

### Standardization process for research personnel

Blinded evaluators undergo a standardization process prior to administering the JTHFT and the FMA. Personnel are provided with online training videos for both evaluations and given administration and scoring guidelines. After practicing with lab personnel, each evaluator is videotaped performing the JTHFT and FMA with a stroke survivor. The evaluators then perform a self-assessment using a standardization scoresheet that outlines performance and scoring guidelines for each evaluation. Once performance accuracy of at least 90% is achieved on self-assessment, the video is then scored by a previously standardized evaluator from the alternate site. Critical issues or discrepancies are discussed. Successful completion of the standardization processes requires a performance accuracy of at least 90% on both the self-assessment and alternate site assessment. Blinded evaluators are also proficient in using the Kinereach system and complete on-line certification of the National Institutes of Health Stroke Scale. Standardization procedures are repeated every 6-months.

### Randomization method

To determine sample size, we used a power analysis program, SOLO, and computer simulation. SOLO permitted us to vary effect sizes and determine the test's power for a fixed group size. The simulation permitted us to use our pilot data to generate multivariate normal data; fit a model with fixed effects for track, test, and track by test interaction; conduct tests to include those for the linear contrasts; and record the p-value and associated effect size. The pilot data was particularly useful because the participants were chosen so that they would span the expected impairment levels, and included right- and left-hemisphere-damaged participants. Based on our primary outcome measures (Barthel, FIM-motor, Abilhand, UEFM, JTHFT) the proposed sample size of 60 participants per group provides adequate power (> = 0.80) to assess each prediction when the effect size (Cohen's f) is 0.35 or greater. Cohen suggested that researchers consider f = 0.1 as a small effect, f = 0.25 as a medium effect, and f = 0.4 as a large effect.

The study statistician then used a Stata-contributed program, alloc, to prepare separate block randomization lists for female and male participants, separately at the Pennsylvania State University (PSU) site and the University of Southern California (USC) site. The use of gender- and site-specific block randomization schemes helps to ensure that research team members who interact with study participants do not know which condition a participant will receive prior to being randomized to the condition. The program uses a variable block randomization scheme that increases the difficulty of an individual using prior assignments to guess the condition to which the current participant will be assigned. All of the assigned participants are randomized to either the experimental condition, VRMT arm intervention or the control condition, conventional paretic-arm intervention. This experimental study design has one between-subjects factor (condition), and two within-subject factors: (time) and the condition x time interaction.

### Intervention: VRMT–Ipsilesional limb

The intervention is designed to be delivered 3 times per week for a duration of 5 weeks (15 visits). The 15 total intervention visits may be compressed to a minimum of 4 weeks and extended to a maximum of 7 weeks in order to accommodate participant schedules. If participants miss more than 3 training visits at a time (i.e., 1 week of intervention), the study team will discuss how to proceed on a case-by-case basis during the weekly team meeting. Missing data points in the testing and intervention sessions will be accounted for during data analysis (see “ missing” in data analysis section for details).

**Fig. 4:**
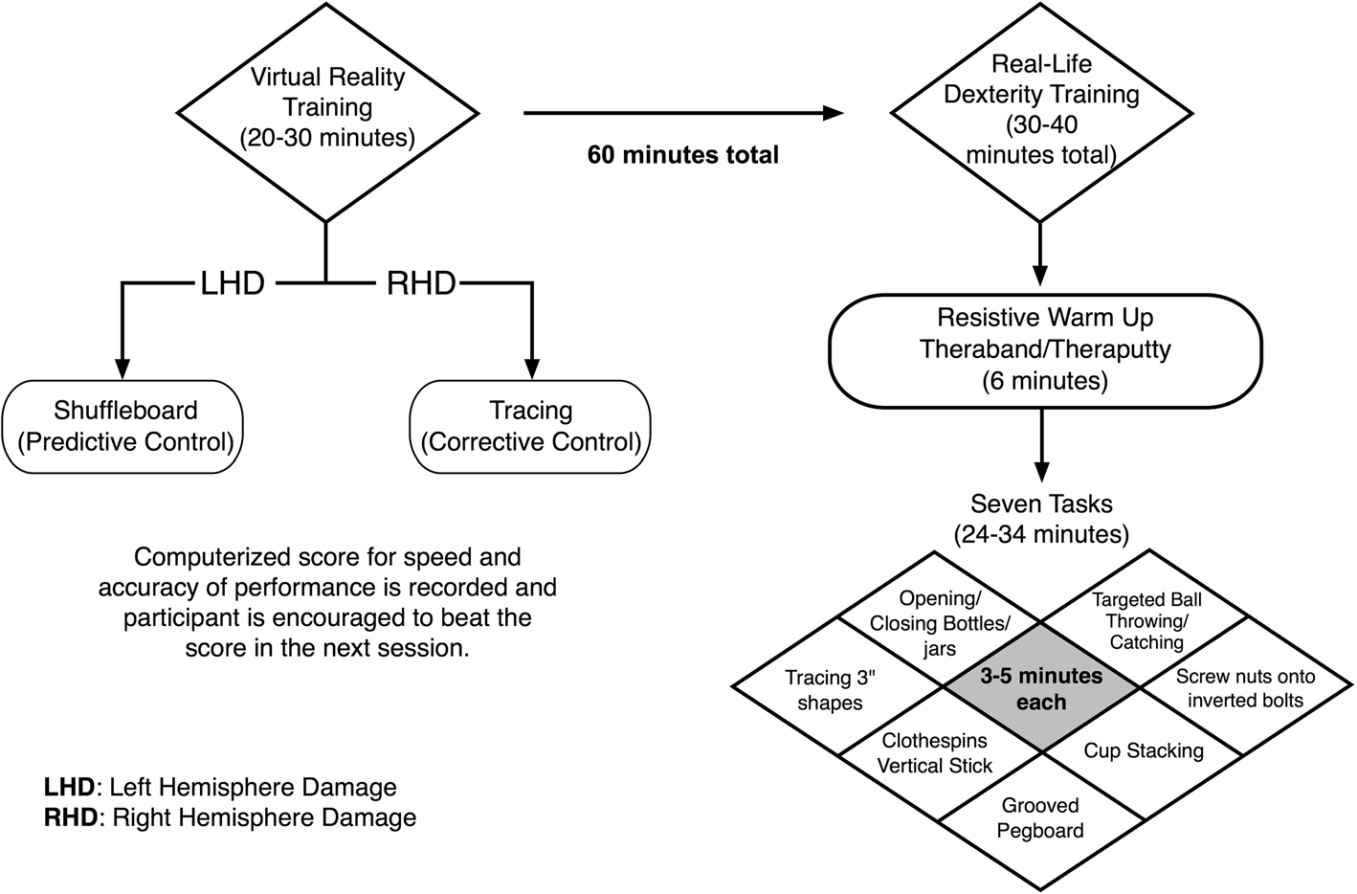
Ipsilesional arm therapy session activities for the experimental group

Each training session consists of two components: 1) virtual reality (VR) games on the Kinereach system that target right- or left-hemisphere-specific motor deficits, depending on which hemisphere is damaged and 2) dexterity training, which does not depend on lesion side (see Fig. 4). The VR session is 20–30 min long, and the dexterity training session is approximately 30–40 min long in order to ensure a total of 60 min of training/session. The VR component involves training on a game designed for the Kinereach motion tracking system, which uses the TrakStar (Northern Digital Inc.), along with two six DOF magnetic sensors to record limb position and orientation data at 116 Hz. Participants are seated in front of the Kinereach, and the sensors are attached to their ipsilesional upper arm and hand. Participants undergo one of two different types of VR training, depending on side of lesion.

For the first 20–30 min of training, participants practice virtual tasks adapted to target the motor control deficits associated with the damaged hemisphere. LHD participants practice virtual shuffleboard, which focuses on predictive aspects of trajectory control, while RHD participants practice a virtual tracing game that focuses on feedback-mediated control. A score is kept at the end of each session and shared with the participant each session as a motivation to beat that score at the next session.

Participants that have a pacemaker or are otherwise unable to receive Kinereach training (due to mobility, discomfort) complete a modified equivalent task outside of the Kinereach system during the virtual reality component of the ipsilesional arm training session. To emulate the shuffleboard task, we use a cornhole-type game where participants sit in front of a table and throw a beanbag into a hole placed on the other side of the table. The distance of the hole is set by determining the full range of elbow extension that the participant is able to comfortably produce. Participants are asked to throw the beanbag with the elbow raised to shoulder level and produce full extension of the elbow at the end of the throw, similar to the motion that occurs during the shuffleboard task on the Kinereach. A score is kept at the end of each session and shared with the participant each session as a motivation to beat that score at the next session. To emulate the tracing task, we ask participants to sit in front of a table and trace shapes placed on a tracing board in front of them. We instruct participants to keep the elbow raised and hold a highlighter between their fingers, perpendicular to the table to emulate the limb position during the Kinereach tracing task. The traces are the same as those used on the Kinereach version of the task. A score of the number of traces completed is recorded at the end of each session.

This VRMT component of practice is followed by 30–40 min of ipsilesional hand manipulation training using a variety of objects geared to improve speed and accuracy. Each task is practiced for approximately 4–6 min, and the score is recorded as a ‘target’ to beat in the next session, providing motivation for improvement and progression within and between sessions. Verbal motivation is also given during the entire session by telling participants to encourage the participants to perform tasks as quickly and accurately as possible.

### Control comparison intervention—contralesional limb

**Fig. 5:**
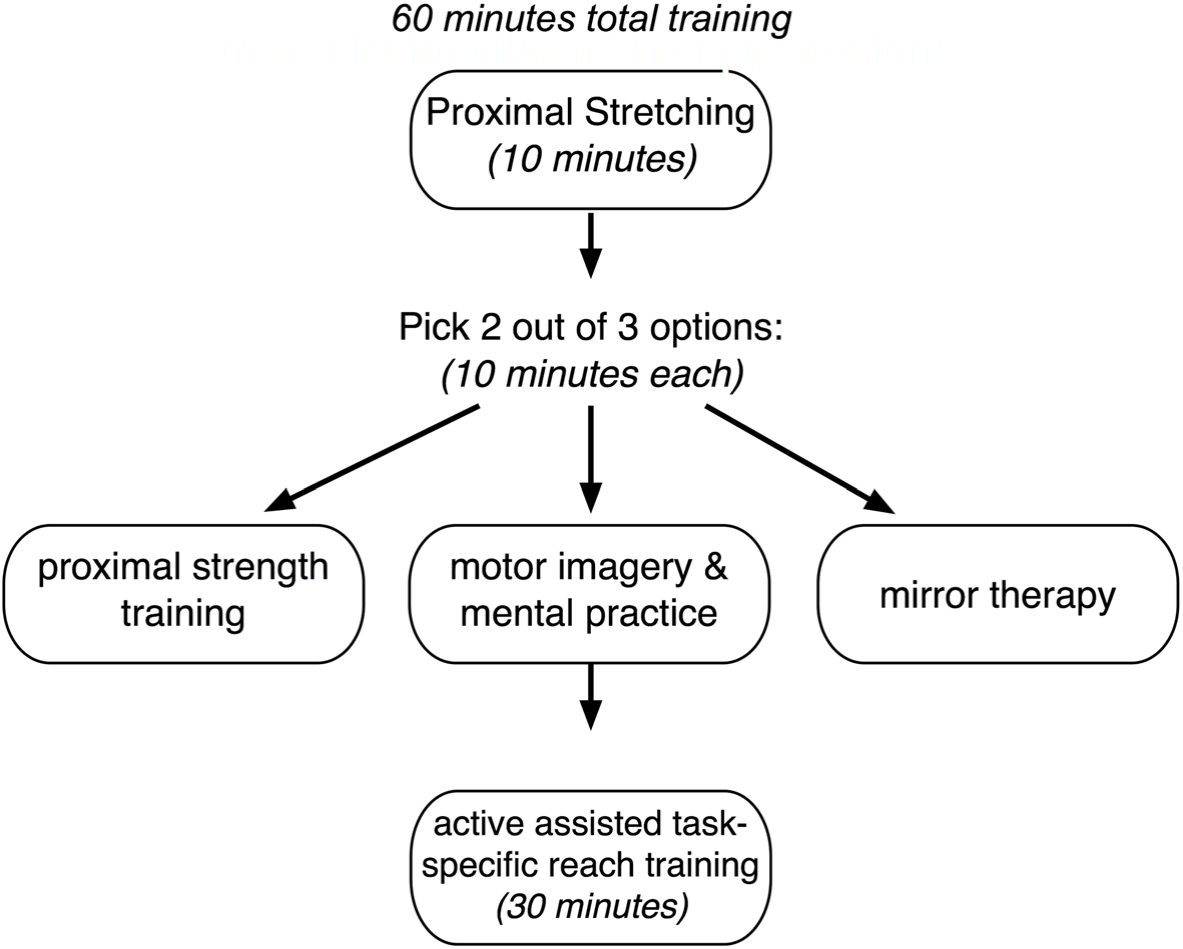
Contralesional arm therapy sessions for participants randomized to the control group

The control comparison intervention is designed based on the best-practices framework for arm recovery post stroke developed by an international group of clinicians and researchers in post-stroke rehabilitation [47]. Each session lasts approximately 60 min and focuses on the contralesional (more impaired) arm (see Fig. 5). The session begins with 10 min of passive range of motion, gentle stretching, and proximal weight bearing exercises that are performed in order to help relax spasticity (if present) and prepare the proximal muscles for participation in activity and decrease risk of musculoskeletal injury. This is followed by performance of two out of three recommended therapies, chosen by the therapist, each lasting about ten minutes: 1) proximal strength training exercises, 2) motor imagery and 3) mental practice, and/or mirror therapy. Lastly, thirty minutes of active assisted task-specific arm reach training in various directions is performed.

Proximal strength training exercise consists of active range of motion exercises of the contralesional arm with resistance if tolerated. Resistance is in the form of Therabands or manual resistance from the clinical interventionist. If resistance is not tolerated, active range of motion exercises are performed free standing. For motor imagery and mental practice, participants are seated in a comfortable position with both feet flat on the floor, upright posture, and hands in a relaxed position with eyes open or closed. Examples of motor imagery activities are washing hands, folding laundry or a scenic walk. Mirror therapy is performed using a midsagittal mirror that reflects the ipsilesional arm. The participant is seated comfortably at a table with both forearms resting on the table. The contralesional arm is placed inside or behind the midsagittal mirror box so that it is no longer visible to the participant. The participant performs slow movements at the wrist and hand joints with both limbs while looking in the mirror at the reflected ipsilesional arm. At the end of each session, the interventionist documents the exercises done and the duration, the scenario(s) used for motor imagery, and any subjective reports or concerns from the participant, such as pain or discomfort.

### Standardization process for the interventions

All interventionists undergo a standardization process for the experimental and control comparison interventions. Personnel are provided with online training videos and in person instruction demonstrating a typical session for both the experimental and control comparison intervention. Intervention administration guidelines and scoring instructions are also provided. After sufficient practice with other study personnel to feel confident to perform both the experimental and control comparison interventions, interventionists are then videotaped conducting the physical intervention on an individual stroke survivor. Similar to the standardization process for evaluators, each interventionist provides a self-assessment using a standardization scoresheet with criteria that specify the various treatment components and time allotments for each experimental and control comparison intervention. Once a self-assessment accuracy score of 90% is met, the video is reviewed by a previously standardized assessor from the alternate site using the same standardization criteria scoresheet. Any critical issues or discrepancies are discussed. Successful completion of the standardization process requires a performance accuracy of at least 90% on both the self-assessment and alternate site assessment. Personnel must also demonstrate proficient use of the Kinereach system. Standardization procedures are repeated every 6-months.

### Specific aims and hypotheses

*Specific Aim 1:* To determine whether ipsilesional VRMT in chronic stroke survivors with severe paresis will produce immediate improvements in less-impaired arm motor performance that will generalize to improved functional activities and functional independence to a greater extent than conventional therapy focused on the contralesional paretic arm.

Primary Hypothesis: Unilateral VRMT of the ipsilesional arm will produce functional improvements in less-impaired arm motor performance, assessed through the JTHFT.

Secondary Hypothesis: Conventional therapy focused on the paretic arm should decrease paretic arm impairment, assessed through the FMA, to a greater or equal extent as ipsilesional VRMT training.

Tertiary Hypothesis: The effects of ipsilesional VRMT training will generalize to functional activities, assessed through the Abilhand, and functional independence, assessed through the FIM-motor and Barthel assessments, to a greater extent than conventional paretic arm therapy.

*Specific Aim 2:* To determine whether intervention-induced improvements in the less-impaired arm performance are associated with improvements in hemisphere-specific reaching kinematics.

Hypothesis: VRMT—induced improvements in performance will be correlated with reductions in hemisphere-specific motor deficits, which are targeted by the VR component of the experimental intervention.

*Specific Aim 3:* To determine whether our experimental intervention (less-impaired arm VRMT) might have detrimental effects on paretic arm impairment, assessed through the FMA and Kinematic measures.

Hypothesis: VRMT dependent improvements in less-impaired arm motor performance, assessed through the JTHFT, will not decrease paretic arm impairment level.

### Statistical analysis

We will fit univariate linear mixed models (LMMs) with single-degree-of-freedom linear contrasts to determine if the data support our predictions. Specifically, we will fit a linear mixed model with fixed effects for condition (intervention, comparison), time (Test), and the condition by time interaction. Linear mixed models provide a more flexible approach to the traditional analysis of variance (ANOVA) model for this repeated measures design. Specifically, LMMs permit us to account for any observed heterogeneity with respect to response variability across time and condition by modeling the mean response and the response’s variance–covariance structure due to the repeated measurements across time. Additionally, LMMs permit each participant to have her or his own initial value (random intercept) and her or his own change pattern across time (random coefficients for an underlying piecewise regression). Finally, LMMs allow us to use covariates to adjust for any potential confounders and can tolerate missing data on the response, which is not true of the traditional ANOVA-based model.

### Analysis plan

This trial is comprised of three specific aims to address the following questions: 1) Will less-impaired arm VRMT in chronic stroke survivors with severe paresis produce immediate improvements in ipsilesional arm motor performance, and generalize to improved functional independence to a greater extent than conventional therapy focused on the contralateral arm? 2) Are improvements in ipsilesional arm performance associated with improvements in hemisphere-specific reaching kinematics? Finally, 3) Does ipsilesional arm VRMT have detrimental effects on contralateral arm impairment?

For the primary aim’s outcome measures, we will fit linear mixed models (LMMs) with single-degree-of-freedom linear contrasts. This model will have fixed effects for condition (VRMT, conventional) and time (see Table 3 for further information on the evaluation endpoints), along with the condition x time interaction. A p-value of 0.05 or less will be used to indicate statistical significance. For the secondary aim, we will examine positional variance at both early (at peak velocity) and late (at end of movement) stage of movement when performing the ipsilesional hand reaching task. Data are low-pass filtered using a 12 Hz zero lag Butterworth filter, prior to differentiating to yield velocity and acceleration profiles. The end of movement is quantified as a tangential velocity minimum, after peak velocity that has an amplitude of less than 15% of maximum velocity for that trial. For the tertiary aim, we will compare FMA scores of those individuals that have received VRMT to those in the control group. We will also use contralateral arm kinematics derived from the contralesional hand work area task to compare between those who have received VRMT and those who have not. As described by Sukal et al. (2007), “work area” is a powerful measure to assess contralesional arm active range in participants with severe paresis [4].

### Missing data

If minimal missing data are found, we will use the flexibility inherent in a linear mixed model to address the missing data. If there is appreciable missing data, we will use multiple imputation (MI) with our linear mixed models and their single degree-of-freedom contrasts. Multiple imputation is a 43 + -year-old method that uses a model-based approach to estimate a plausible value for each missing observation. It has known advantages over earlier approaches such as casewise deletion (i.e., dropping cases with any missing data, which can lead to an appreciable loss of data) and single imputation (e.g., replacing each missing observation with the mean of the observed responses, which cannot provide an estimate of the uncertainty due to this replacement). Specifically, MI is able to provide an estimate of the uncertainty due to replacing each missing observation with a plausible value. It does this by replacing each missing observation with "m" plausible values. More importantly, MI provides a rational basis for estimating the uncertainty associated with a given imputed data set and the total uncertainty due to processing the "m" imputed data sets. Multiple imputation is currently an active area of research, which is not the case for the corresponding mixed effects ANOVA.

### Adverse event monitoring and reporting procedures

An adverse event (AE) is reported in terms of serious (SAE) or non-serious (NSAE) with further classification either expected or unexpected and related or unrelated to study participation. Both SAEs and NSAEs can occur on-site or be reported by the participant after they occur off-site. Expected non-serious events include, but are not limited to, the following: fall with no fracture, dyspnea, open sore or cuts, muscle soreness or pain that persisted for more than 48 h, shoulder pain that limited study participation, excessive blood pressure response that requires treatment discontinuation for the day, dizziness, deep venous thrombosis. Offsite AEs are monitored by study personnel at the baseline evaluations, the mid-point of the intervention and each follow-up post-test. Upon notice or report of any AE, serious or non-serious, study personnel immediately report AEs to both PIs and the clinical site coordinator. In the event of SAE occurrence on-site, study procedures are immediately stopped and reported to the medical monitor who will liaise with the DSMB. The site PI will determine if offsite AEs need to be reported to DSMB. DSMB will provide all related documentation to the IRB.

SAEs and NSAEs are documented by the study personnel. NSAEs will be reported to the IRB every 6 months. All NSAE events will be monitored by the clinical site coordinator until the AE is resolved or up to 1-month after the end of study participation.

### COVID-related procedures and processes 

In March of 2020, PSU received notice that studies involving direct participant contact with no direct drug or device therapeutic benefit were to be postponed until further notice. In July of 2020, this clinical trial was deemed essential due to the benefits for people living with stroke. Study procedures were re-initiated in September 2020 and closed again in November 2020 due to the surge in COVID-19 cases across the country. In June 2021 procedures for in-person studies at PSU were able to resume on a case-by-case basis.

In March of 2020, USC was notified that clinical research involving direct participant contact must be halted until the implementation of COVID-19 specific safeguards received IRB approval. Safeguards included updated consent forms reviewing the increased risks of COVID-19, health screening, appropriate PPE, sanitation procedures, etc. IRB approval of these added safeguards was obtained in October of 2020, and study procedures were resumed in a limited capacity (i.e., only one participant was seen at a time). In December 2020, due to the rapid increase and spread of COVID-19 in LA county, study procedures were again halted. Study procedures using risk mitigation procedures (i.e. PPE, Masks, Social distancing) were resumed in May 2021, once all study staff at USC were vaccinated.

### Recruitment before and after COVID-19

In order to accommodate the disruption in enrollment caused by the global pandemic, planned recruitment (120 participants) has been adjusted to account for the period of time when the laboratories were not accepting new participants. The study is now projected to continue recruitment an additional year, with the study ending in May of 2024.

### Data management and quality

Each site is responsible for site-specific data collection. Data management includes storage, security, confidentiality, and data entry. Hard copies of data CRFs are stored in secure, locked cabinets at each site, accessible only by study personnel. Data from CRF documents are input and stored using the REDCap web application, managed by Penn State. Kinematic data collected using the Kinereach Device is de-identified and uploaded to the shared PSU Box server to be analyzed by members of the research team at PSU. Monthly backups of all data on the shared Box drive are performed by a member of the PSU site onto an encrypted external hard drive secured at PSU.

### Quality control procedures

This study is registered with ClinicalTrials.gov (Registration ID: NCT03634397). A Manual of Procedures (MoP) was developed and stored on the shared Box server for accessibility to all study personnel at both sites. The MoP is a living document that includes a log of all changes by date and version number. The MoP includes details of all study procedures including but not limited to the study protocol, data collection, CRFs, data storage, data analysis, and dissemination procedures. All new personnel are required to review the MoP. In person meetings are held bi-annually (pending Covid-19 restrictions) to ensure all lead personnel are similarly trained at both sites. Standardization procedures ensure personnel at both sites similarly administer the interventions and/or evaluations for study participants.

### Study organization and management

This study is under a Multiple PI leadership team of Robert Sainburg (Primary Site, PSU) and Carolee Winstein (Secondary Site, USC). The PIs have put together an experienced team of basic and translational scientists at each site, including site-specific administrative personal such as the clinical site coordinator (CSC), interventionist, blinded assessor, research assistant and back-up staff who can fill in for staff who are not available. The primary contact PI is Dr. Robert Sainburg, PhD, OTR, who is responsible for the overall conduct of the study, along with the development of all protocols for interventions and outcomes used for assessment. He holds overall fiscal responsibility for the study, communicates with the Data Safety and Monitoring Board, and leads the steering committee in collaboration with Co-PI Winstein.

Co-PI Dr. Carolee Winstein, PhD, PT, FAPTA, brings extensive clinical trial expertise to the team. She assists Dr. Sainburg with distributed PI responsibilities across both sites including the development and continuous updating of the Manual of Procedures (MoP). She oversees the implementation of the study at the USC site, including all hiring, administrative and clinical responsibilities and she participates in weekly team meetings pertaining to the conduct of IPSI.

The Data Safety and Monitoring Board (DSMB) is independent from the study team and periodically examines the study procedures and data to ensure safety and ethical study execution. PSU members of the board include the study biostatistician, a physical medicine and rehabilitation physician, and the director of the office of patient-oriented research. USC members of the board include a neurologist and a physical medicine and rehabilitation physician. The DSMB is responsible for oversight of the study, including interim analyses, quality control, and participant safety. All serious adverse events and participant withdrawals, will be reported to the DSMB immediately. All NSAEs are reported every six months or annually for review. Based on their review, the DSMB has the opportunity to provide recommendations regarding protocol modification if deemed necessary. They also provide annual updates to the Penn State Institutional Review Board, the primary IRB overseeing the study, and these updates include a study summary specific to each site. The physical medicine and rehabilitation physician at PSU and the neurologist at USC serve as Medical Monitors at their respective site and they are responsible for the continued monitoring of each participant’s health and safety. They immediately review any SAE reported at their local site and determine appropriate action.

The project manager (CM) reports directly to both PIs and is responsible for implementation of the protocol across both sites. Specifically, CM ensures that both sites are in compliance with the primary IRB (PSU), all interventionists and assessors are standardized, and all information in the MoP is appropriately updated when necessary or called for by either of the two PIs.

Within each site, a team of blinded assessors, interventionists, and research assistants will ensure that the study progresses appropriately. These individuals are led by a CSC, who reports to the site PI and RCT project manager. The CSC is responsible for administrative oversight of their site under the direction of the site PI. They also assist the project manager in training all members of the study team, along with participant recruitment, oversight of data entry, IRB compliance, and ensuring timely standardization of all interventionists and assessors.

Within each site’s study team, several clinical research assessors are responsible for administering all assessments. These individuals are blinded to each participant’s group assignment. They have been standardized by the CSC and conduct all baseline and post-test evaluations. There are also several interventionists, composed of two groups: contralesional (control) arm and ipsilesional (experimental) arm interventionists. These individuals are standardized by the CSC and are responsible for delivering the appropriate intervention according to the outlined research protocol. Given that the experimental and control comparison intervention are radically different in target and content, there is no concern about contamination. As such, a single interventionist can be tasked with administration of both intervention protocols. Lastly, research assistants at each site make a vital contribution by assisting with ongoing administrative duties, including, but not limited to: participant scheduling and compensation, data entry, documentation logs, set-up/clean-up of intervention sessions, data backup, and clerical duties. At the same time, our research assistants are learning about best practice clinical research skills that bode well for future careers in healthcare and clinical research.

The study statistician is responsible for the development of the randomization list and the development of all programs for interim and final data analyses. This individual will participate in manuscript writing of the primary outcome paper. This study also has a neurologist serving a consultant role, in order to assist with the review of MRIs for each potential participant. This individual ensures that potential participant lesions coincide with inclusion criteria.

The publications committee consists of the two PIs and the study biostatistician. The primary purpose of this committee is to facilitate and coordinate publication of the findings of the IPSI project to ensure that these publications are of high quality, both scientifically and ethically. The publications committee is responsible for reviewing and approving (1) overall dissemination plans of the administrative, clinical, and data management teams within the trial, (2) planned journal submissions, and (3) final versions of submitted publications, including abstracts. The publications committee will also be responsible for mediating disagreement among the team regarding the form or scope of the publication, such as authorship. Importantly, the publications committee has taken an inclusive perspective and will provide guidance to junior members of the IPSI team in all dissemination efforts. Plans for any potential publications emerging from the IPSI study must be vetted by the publication committee prior to development through a proposal process that includes the lead, writing team, purpose, overall content, analysis plan, target journal and timeline.

## Discussion

The IPSI trial will provide unique information about the sensorimotor deficits of the ipsilesional arm in chronic stroke survivors with severe motor impairments, including how these deficits can potentially be remediated to produce immediate improvements following training in functional independence. Further, this phase II randomized control trial (RCT) is innovative in that it uses kinematic analysis in a virtual reality environment to directly measure and target ipsilesional arm deficits in chronic stroke survivors with severe deficits. This targeted remediation is hemisphere-specific and addresses mechanistic deficits that arise from a fundamental understanding of unique hemispheric specialization of motor control emergent from each hemisphere. This trial’s approach is novel when compared to usual clinical practice, which largely ignores treatment of the ipsilesional arm in those with severe hemiparesis, except in the context of compensatory strategies and bimanual training paradigms. Current in-patient therapy for those with severe paresis is driven toward accomplishing functional gains with little time and thereby promoting task-specific training of essential ADLs performed solely with the ipsilesional (less-impaired) limb, which we argue is limited in scope. Given that our experimental group receives ipsilesional arm therapy, of which portions are unique to the damaged hemisphere, and our control group receives an alternative appropriate therapy focusing largely on the contralesional arm, we can compare the functional improvements between these treatments and determine if ipsilesional arm training according to the IPSI protocol improves motor control deficits and increases functional independence. If this training protocol is found to be beneficial for chronic stroke survivors with severe paresis, these results should be followed up with a phase III pragmatic trial to determine if the beneficial effect can be generalized to the acute stroke survivor. These findings could inform current clinical rehabilitation practices and further clinical research in those with severe motor impairments.

## Data Availability

The de-identified datasets generated and/or analyzed during the current study will be made available following study completion in the ScholarSphere repository, [https://scholarsphere.psu.edu/], and from the corresponding author upon reasonable request.

## Abbreviations

RHD: Right hemisphere damage
LHD: Left hemisphere damage
VRMT: Virtual reality manipulation training
JTHFT: Jebsen-Taylor hand function test
FMA: Fugl-Meyer Upper Extremity Assessment
FIM: Functional Independence Measure
VR: Virtual reality
MoP: Manual of Procedures
CSC: Clinical site coordinator
MI: Multiple imputation
AE: Adverse event
SAE: Serious adverse event
NSAE: Non-serious adverse event
LMM: linear mixed model
CRF: Case report form
